# Two-Port Laparoscopic Adrenalectomy in Dogs

**DOI:** 10.3390/ani12212917

**Published:** 2022-10-25

**Authors:** Francesco Collivignarelli, Amanda Bianchi, Andrea Paolini, Massimo Vignoli, Paolo Emidio Crisi, Ilaria Falerno, Andrea De Bonis, Martina Rosto, Roberto Tamburro

**Affiliations:** Faculty of Veterinary Medicine, University of Teramo, 64100 Teramo, Italy

**Keywords:** laparoscopic adrenalectomy, laparoscopy, dog, two ports, adrenal tumor, surgery, complications

## Abstract

**Simple Summary:**

An adrenal mass represents the primary indication for adrenalectomy. Laparoscopic adrenalectomy is a minimally invasive surgical technique that minimizes pain and improves patient recovery. The traditional technique involves the use of a single or multiple port (three or four). The objective of the study was to evaluate the feasibility and complication rate of two-port laparoscopic adrenalectomy to remove adrenal masses smaller than 4 cm in diameter. In total, 16 dogs with adrenal masses were enrolled in the study and clinicopathologic data were harvested from the medical records. The dogs were placed in sternal recumbency with cushions positioned under the sternum and pubis to simulate the quadrupedal station and displace the abdominal viscera. In all cases, the procedures were feasible with two ports. In five cases, capsule rupture occurred, and all were adrenal gland carcinomas.

**Abstract:**

The gold-standard treatment for functional tumors is adrenalectomy, and the procedure can be either open or laparoscopic. Laparoscopic adrenalectomy (LA) is a minimally invasive technique designated for small–medium-sized adrenal tumors without vascular invasion. LA is routinely performed using three or four ports with the patient in sternal or lateral recumbency. The aim of the study was to evaluate the feasibility of LA with two ports in order to reduce invasiveness and improve patient recovery. In total, 16 dogs with adrenal tumors were included in the study and the two-port technique was performed. Adrenalectomy was performed based on the presence of hypercortisolism in thirteen cases, whereas, in three cases, adrenalectomy was performed in the absence of evidence of cortisol production. Thirteen cases were carcinomas and three were adenomas of the adrenal gland; furthermore, twelve were on the left side and four were on the right side. Capsule rupture occurred in five cases. After performing the technique in all cases, no additional ports or laparotomy conversion occurred. Based on the authors’ experience, laparoscopic adrenalectomy can be performed with two ports.

## 1. Introduction

Adrenal tumors (AT) represent 1–2% of all canine tumors; furthermore, they can be divided into two groups regarding their hormone-producing abilities: functional or not functional [1]. The excess secretion of cortisol, catecholamines, aldosterone, progesterone, and steroid hormone precursors has been documented in dogs and cats [2]. The most common functional adrenal tumors in dogs secrete cortisol or catecholamines, and they are mostly cortical adenomas, cortical adenocarcinomas, and pheochromocytomas [3]. Bilateral adrenal tumors rarely occur in dogs and are more frequently unilateral [4]. Treatment for adrenal masses depends on the tumor size, hormone production, vascular invasion, and the patient’s clinical condition [5]. The surgical removal of the adrenal gland to remove the source of the excessive production of hormones is the gold-standard treatment for functional adrenal masses [3]. A study suggested that malignancy should be strongly suspected in any mass ≥2 cm in the dorsoventral (DV) dimension measured from a longitudinal ultrasound image or with evidence of vascular invasion [4]. A surgical approach can be open or minimally invasive. Laparoscopic adrenalectomy (LA) is a minimally invasive procedure that is an alternative to conventional laparotomic approaches in both human and veterinary medicine for adrenal masses of small–moderate sizes [6]. Vascular invasion currently represents the application limit for a minimally invasive technique. The invasion of the caudal vena cava requires a cavotomy during an open adrenalectomy (OA) to remove the thrombus [7,8]. Reports from previous studies have documented that a laparoscopic approach lasted a shorter time than open surgery [3,9]. In addition, LA was associated with lower complication rates, less pain, and a faster functional recovery compared with laparotomy. LA has been documented in veterinary medicine since 2008 [10], and different approaches have been described in the last decade [9,11,12]. The surgical procedure can be performed with dogs in lateral or sternal recumbency using a single or multiple ports [13]. The lateral recumbency is feasible and without intraoperative complications [10]; however, the sternal recumbency also has advantages, allowing for the gravitational displacement of the abdominal organs, better visualization of the adrenal gland, and shorter surgical times than conventional laparoscopy or open adrenalectomy [11]. Currently, in veterinary medicine, three or more ports are routinely used for laparoscopic adrenalectomy [2]. A reduction in the number of ports could improve the patient’s recovery, resulting in less pain [14].

The aim of the study was to evaluate the feasibility of a two-port laparoscopic adrenalectomy. The purpose was to develop and describe the technique, as well as its relative complications, in dogs with a unilateral AT. 

## 2. Materials and Methods

The study was retrospectively approved by the ethics committee of the University of Teramo (Prot. n. 6127 28/2/2022). All of the owners signed an informed consent form about the anesthetic and surgical procedures and related risks.

### 2.1. Patient Selection and Data Collection

The inclusion criteria for the unilateral two-port laparoscopic adrenalectomy were dogs with a unilateral adrenal mass size <4 cm in DV measured from a longitudinal ultrasound image. The clinical suspicion of hypercortisolism was based on the clinical signs, physical examinations, and laboratory abnormalities, and the diagnosis of syndrome was confirmed with a low-dose dexamethasone suppression (LDDS). Those dogs with cortisol-secreting tumor were treated with trilostane (Vetoryl^®^; Dechra, Torino, Italy) for 3 to 4 weeks before surgery. Each patient underwent blood pressure measurement, blood type determination, an abdominal ultrasound, and whole-body computed tomography (CT) pre- and post-contrast investigations with no evidence of vena cava invasion. Following the LA, a histopathological examination of the masses was performed. Data on the signalment, patient history, clinical signs, laboratory abnormalities, tumor size, the adrenal gland involved, surgical time, intraoperative and postoperative complications, hospitalization time, histopathological diagnosis, and clinical outcomes were collected.

### 2.2. Surgical Procedure

The anesthetic protocols were not standardized but were selected based on patient exigence. All dogs were premedicated through a 20G or 18G (Jelco^®^; Smiths Medical, Latina, Italy) vein catheter (IV), with methadone (Semfortan^®^; Dechra, Torino, Italy) ranging from 0.1 to 0.3 mg/kg. Dogs were induced with propofol (IV) (Propovet^®^; Zoetis, Roma, Italy) ranging from 4 to 6 mg/kg and maintained with isoflurane in a 50% mixture of air and oxygen. Of the treated dogs, nine received systemic analgesia with fentanyl (Fentadon^®^, Dechra, Torino, Italy) at 5 mcg kg/h with an attack bolus of 2 mcg/kg, and the remaining dogs received an epidural injection of 0.2 mL/kg ropivacaine 0.2% (Ropivacaine hydrochloride^®^, Molteni, Firenze, Italy) and morphine 0.1 mg/kg (Morphine hydrochloride^®^, Molteni, Firenze, Italy). Clinical and instrumental parameters were recorded every 5 min: heart rate (HR); respiratory rate (fR); oxygen saturation (Sp02); and invasive systolic, mean, and diastolic pressures (SAP, MAP, and DAP). All surgical procedures were performed by one expert surgeon (F.C.). The operative time was recorded from the skin incision to the complete skin suture application.

The dogs were clipped from the eighth rib to the paralumbar fossa, and from the spiny processes of the lumbar vertebrae to the ventral midline. An intravenous 22 mg/kg dose of cephazolin (Cefazolina Teva^®^, Teva Italia S.r.l., Milano, Italy) was administered 30 min before the skin incision and repeated every 90 min during the procedure.

**Figure 1:**
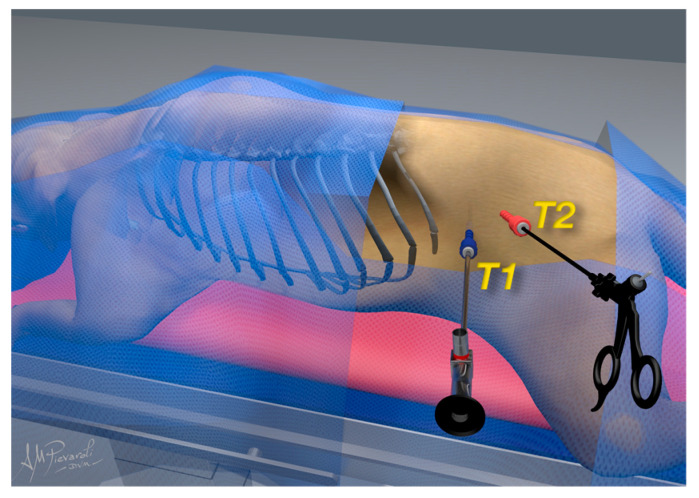
Positions of T1 and T2 are represented in the figure. T1 is behind the last rib and T2 is in the paralumbar fossae.

The dogs were placed in sternal recumbency with cushions under the abdomen to simulate the quadrupedal station and to separate the adrenal gland from the surrounding organs; additionally, the surgical table was tilted by 30 degrees as previously described [11]. One cushion was placed between the pelvic limbs extending no further cranial than the pubis to support the pelvis, and the other cushion was placed under the sternum to elevate the chest. The ports were placed along an ideal line extending from the 13th rib to the iliac crest. Two surgical approaches were performed using a modified Hasson technique with the Airplasma^®^ device (Airplasma^®^, Onemytis®, Alessandria, Italy) [15], with dimensions of 6 and 11 mm, respectively. The skin and the subcutaneous tissue were incised with the device, and the muscle fascia was exposed and raised with a clamp and then incised with Airplasma^®^ together with the muscle layer and peritoneum. In the first surgical access, behind the last rib, a 6 mm cannula (T1) (62160GBK, Karl Storz endoscopy, Verona, Italy) was placed. The abdomen was inflated with CO2 (Endoflator^®^ 50, Karl Storz endoscopy, Verona, Italy) until an intra-abdominal pressure of 8–10 mm Hg was achieved. The inflation was adjusted according to the dog’s size and physiologic variables. An 11 mm second port (T2) (60123 EV, Karl Storz endoscopy, Verona, Italy) was placed in the paralumbar fossae (Figure 1).

**Figure 2:**
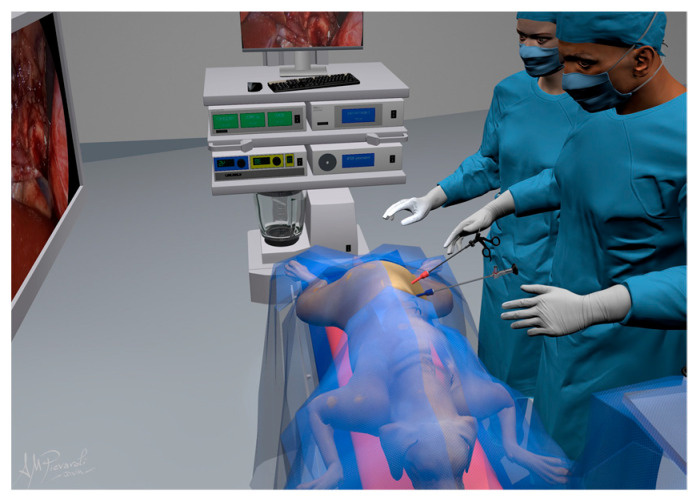
Instruments and dog positions are represented in the figure. The surgeon position is on the adrenal tumor side.

In T1, a 5 mm 30° laparoscope (Hopkins^®^ II 62046 BA, Karl Storz endoscopy, Verona, Italy) was inserted, and a visual exploration of the abdomen was performed to evidence metastatic deposits or signs of other diseases or neoplasms. In T2, a sealing device LigaSure™ V (LS1500, Covidien, Milano, Italy), a laparoscope Babcock forceps (33310A, Karl Storz Endoscopy, Verona, Italy), or a laparoscope Kelly forceps (66322ML, Karl Storz Endoscopy, Verona, Italy) was inserted. A video monitor was positioned in front of the surgeons on the dorsal side of the dog (Figure 2).

**Figure 3:**
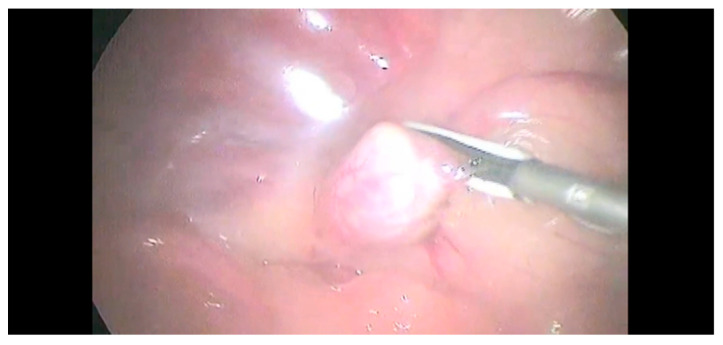
The circumferential dissection of the adrenal gland was achieved using a 5 mm sealing device.

Anatomic differences between the right- and left-side adrenal glands required a different type of dissection. Babcock forceps, Kelly forceps, and the sealing device were introduced in T2 to obtain a better exposure of the right-side adrenal gland. The circumferential dissection of the adrenal gland was achieved using the 5 mm sealing device (Figure 3).

**Figure 4:**
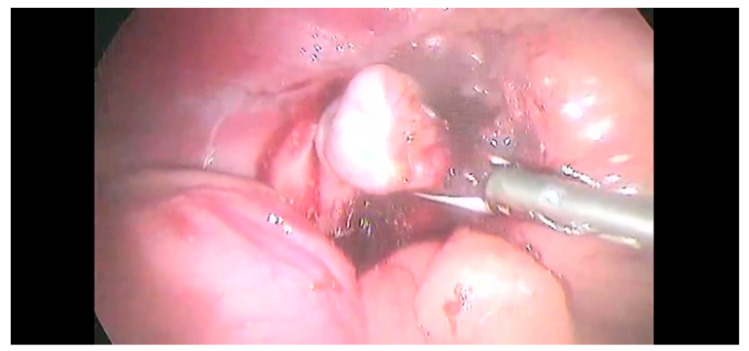
The phrenicoabdominal (PA) vein and artery were identified and sealed using a sealing device.

On the left side, the dissection started with the blunt incision of the peritoneum over the caudal margin of the tumor using the Kelly forceps or, alternatively, the tip of a vessel sealer. The dissection was continued cranially until the phrenicoabdominal vessel was evident, then, it was sealed and sectioned with LigaSure™ (Figure 4); however, laparoscopic hemoclips (Endo clip II ML, Tyco Health- care, Milano, Italy) were sometimes used in addition to the vessel-sealing device.

**Figure 5:**
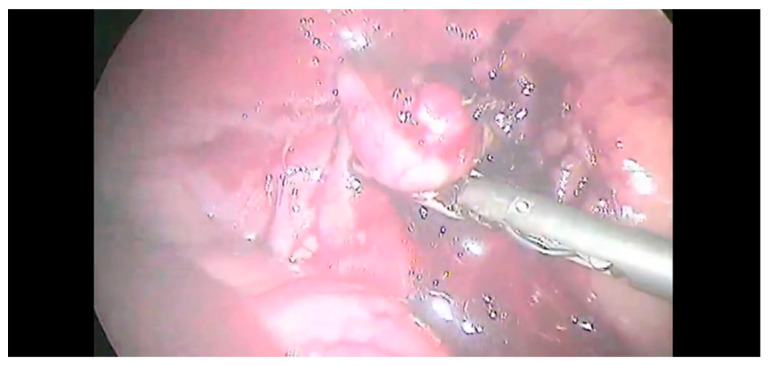
Complete dissection of the adrenal gland.

The adrenal gland was dissected from the fibrous tissue attached to vena cava. The risk of capsule rupture was limited through the careful manipulation of the gland and surrounding fat. The capsule of the right-side masses was continuous with the tunica externa of the vena cava; hence, the dissection was initiated between the mass and the vena cava. Then, the dissection was continued as described for left-side lesions. Once the dissection was completed (Figure 5), the adrenal gland was removed with a surgical glove or retrieval bag to minimize the neoplastic cells spared from the T2.

After a visual inspection of the peritoneal space, the abdomen was decompressed, and the laparoscopic accesses were routinely closed. All of the masses were submitted for histopathological examination to assess the tumor type.

### 2.3. Postoperative Care

After surgery, the patients were recovered in a critical care unit (ICU), and blood glucose, electrolytes, invasive blood pressures, and electrocardiograms were strictly monitored. In those dogs with cortisol-producing adrenal tumors, dexamethasone 0.05 mg/kg EV was administered intraoperatively and then 8 h apart. The dose of dexamethasone was decreased at 0.03 mg/kg every 12 h until the dog could safely receive oral medication. After that, glucocorticoid supplementation was switched to oral prednisolone at 0.5 mg/kg twice daily for 3 days and the dose was tapered to 0.1–0.2 mg/kg after 2–4 weeks. Adrenal reserve, and, subsequently, the need for corticosteroid replacement, was assessed every 2–3 weeks by the ACTH stimulation test. The supplementation was discontinued after an ACTH stimulation test within normal limits.

Heparin constant rate infusion (CRI) was administered starting at 10 IU/kg/hour, adjusting the dose to achieve 1.5 times the baseline partial thromboplastin time (PTT). At the discretion of the attending clinician, the dogs were discharged with subcutaneous heparin at the dosage of 150–250 IU/kg every 6 h or oral clopidogrel at 1–3 mg/kg once a day for 7–14 days.

### 2.4. Complications

Surgical complications were recorded. Possible intraoperative complications included: bleeding, capsule rupture, iatrogenic damage to abdominal organs, hypotension, hypertension, arrhythmias, hypoxemia, and death. Possible postoperative short-term complications included: gastrointestinal disorders, pancreatitis, acute renal failure, pulmonary thromboembolism (PTE), disseminated intravascular coagulopathy (DIC), and death within 14 days PO. The information collected during mid and long-term follow-up included: recurrence of tumor or clinical signs, death related to adrenal tumor surgery, death for reasons unrelated to the tumor, and other disorders or neoplasms. Data were collected via direct contact with owners or referring veterinarians.

### 2.5. Histopathology

The samples were fixed in 10% formalin for 48 h; then, they were cut and strained with hematoxylin and eosin for microscopic examination. Differentiation between adenoma and carcinoma was based on the histological criteria described by Labelle et al. in 2004 [16]. A trabecular growth pattern, peripheral fibrosis, capsular invasion, necrosis, and/or hemorrhage were considered as histological characteristics of carcinomas. Hematopoiesis, fibrin thrombi, and cytoplasmic vacuolation were associated with adrenocortical adenomas.

### 2.6. Statistical Analysis

The statistical analysis was performed with Jamovi computer software Version 1.6 (Sydney, Australia). Data on the surgical time and adrenal gland size were screened for normality using Shapiro–Wilk tests. The results were expressed as the mean and standard deviation for data with normal distribution, or the median and range for data without normal distribution. The surgical times between the left- and right-side tumors were expressed as the mean and standard deviation and were compared using the Student’s t-test. The complication rates were expressed as percentages, and a Fisher’s exact test was performed to compare complication rates in the left- and right-side tumors. The significance level was set at *p* value ≤ 0.05.

## 3. Results

### 3.1. Clinical Study

Sixteen unilateral laparoscopic adrenalectomies were enrolled in this study (Table 1). Five adrenal masses were incidental findings during an ultrasound imaging examination, and eleven were found during an ultrasound examination prescribed based on the clinical signs and laboratory abnormalities. Twelve adrenal masses were on the left side, and four were on the right side. Eleven dogs presented at the hospital with one or more clinical signs: polyuria/polydipsia (PU/PD) in ten cases, alopecia in five cases, calcinosis cutis in four cases, polyphagia in two cases, hematuria in two cases, stranguria in one case, and thin skin in one case (Table 1). In 13 out of 16 dogs, the LDDS test was suggestive of hypercortisolism. No dogs showed alterations of the pituitary gland upon CT examination.

The mean surgical time was 70.5 ± 24.7 min. The mean surgical time was 67.4 ± 22.2 for the left-side adrenal glands, and 79.8 ± 33 for the right-side adrenal glands; however, the difference was not statistically significant.

### 3.2. Complications

The complications were reported in Table 2. Capsule rupture was a major intraoperative complication that occurred in five cases (27.7%); furthermore, postoperative complications included periportal cellulitis, which occurred in five cases (27.7%), and mild peritonitis, which occurred in one case (5.5%). Comparisons between the incidences of capsule rupture in the LS and RS tumors were not statistically significant. All five capsule ruptures were carcinomas. No patients required conversion to laparotomy, third-portal creation, or position changing. No patient developed pancreatitis, acute renal failure, pulmonary thromboembolism, or died postoperatively within fourteen days.

### 3.3. Outcomes

Six dogs are alive at the drafting stage of this manuscript. One dog was lost at the follow up. Two dogs died of metastatic disease 3 and 5 years after surgery. A total of six dogs died in the period between 4 and 18 months after surgery. The data are summarized in Table 2.

### 3.4. Histopathology

Twelve tumors were classified as carcinomas (66.67%), and six as adenomas (33.3%); the data are reported in Table 2. Histologic evidence of neoplastic emboli was observed in the AT tissue of five dogs.

## 4. Discussion

Two-port laparoscopy is less invasive than multiple-port adrenalectomy; furthermore, the literature suggests that a reduced number of ports could minimize soft tissue trauma, improve cosmesis in human patients [14], and reduce postoperative pain in dogs undergoing laparoscopic ovariectomy [17] For this reason, the purpose was to demonstrate the feasibility of developing a novel LA in order to provide alternative and less invasive techniques, with the prospect of improving patient recovery rates and reducing the postoperative pain and hospitalization time. The low incidence rates of the minor postoperative complications were in accordance with the data of previous reports and confirm the rapid recovery of patients undergoing minimally invasive procedures [18,19,20]. The objective of the present study was to evaluate the feasibility of the technique; however, no data of the pain scores were recorded. A comparison between multiple-, two- and single-port adrenalectomies in terms of postoperative pain, recovery time, and hospitalization time is required.

The authors were able to successfully execute all sixteen procedures using the two-port technique for both left- and right-side adrenal tumors. The sternal recumbency avoided operating-table-determined compression on the viscera and improved the visualization field of the adrenal gland and surrounding structures. Gravitation forces allowed for an excellent exposure of the adrenal gland and did not require the use of a second instrument, which simplified the dissection. The use of a single instrument in a small surgical field reduces instrument collisions, leading to an improved dissection of the adrenal gland.

The creation of a third portal, a change in patient position, or the conversion to laparotomy was not necessary in any case. The surgical mean time in the present study was 70.5 min, and recent reports showed mean times of 78 and 90 min using three or more ports, respectively [9,11].

Intraoperative complications were observed in 5/16 (31%) of the procedures, and all of these were related to capsule rupture. The histopathological diagnosis was adrenal gland carcinoma in all of these patients. In the results of previous reports, the percentages were similar, and capsule rupture varied from 22% to 31% of recorded complications [9,11]. The literature has reported significantly higher incidences of rupture in malignant tumors compared with benign, possibly because the carcinoma tissue is extremely fragile [10]; however, the clinical consequences have not been clarified. In the study population, two out of five dogs that experienced capsule rupture died from metastatic disease 3 and 5 years after surgery, two out of five died in the period between 8 and 14 months later, and one dog was lost to follow up. The high incidences of capsule rupture could be a consequence of the high prevalence of carcinoma in the population (13/16 tumors). In previous studies, the prevalence of malignant tumors was significantly lower compared to the present results [4]. In two-port laparoscopic adrenalectomy, the dissection of the adrenal gland involves dissecting the peri-glandular tissues, including the fat pad surrounding the gland. This approach may be less accurate than multiple-portal techniques because there is a lack of instruments dedicated to organ retraction, which is useful for performing dissection. The results of previous studies showed that pheochromocytomas were the second most common adrenal tumor type in dogs [21,22]; however, their tendency toward vascular invasion often does not make them ideal candidates for minimally invasive surgery [23]. No entry complications were encountered during any of the procedures, despite these being the most frequent complications described in laparoscopy [24].

An appropriate case selection is essential in laparoscopic adrenalectomy due to the close anatomic relationship of the glands to large vascular structures and the biological behaviors of these tumors, which cause them to invade vessels and other structures [2]. In this study, case selection excluded patients with vascular invasion, and a TC was performed in all patients to estimate the extent of the tumor in the surrounding tissues and distant organs. The right adrenal gland is more complex to approach surgically, and the gland capsule can be in continuity with the tunica externa of the caudal vena cava in dogs, making the dissection challenging in minimally invasive surgery; however, four successful right-sided adrenalectomies were performed in the present study. The tumor size represents an application limit, and, in the study, masses smaller than 4 cm were included.

No dogs died in the perioperative period. The results of some studies reported that 19% of perioperative deaths were due to pancreatitis, acute renal failure, pulmonary thromboembolism, or cardiac arrest [25]. Currently, adrenalectomy is considered a surgical procedure associated with high mortality rates [26,27]. Recent reports have documented that tumor sizes >5 cm and the presence of metastasis or vein thrombosis had poorer prognoses [28]. When an open approach was used in patients affected by small adrenal tumors without vascular invasion, the short-term survival rate was 92.2% [29]. In a recent study, short- and long-term outcomes were similar when comparing OA and LA in dogs [3]. Articulate instruments are currently available. They are specifically designed instruments with articulated and adjustable branches that allow clinicians to work in small spaces without collisions [30]. In the present study, traditional laparoscopic instruments were used; however, we could speculate that the use of dedicated instruments would allow for a reduction in surgical times and would simplify the dissection of the adrenal gland for less-experienced surgeons. The hypothesis should be confirmed by further studies. All sixteen LAs were performed by an expert surgeon in the minimally invasive field, and this factor contributed considerably to the feasibility of the procedures. Looking at the results, the surgeon’s learning curve is evident. The increased confidence with the technique allowed for progressively shorter operating times, but there seems to be no correlation between the surgeon experience and incidence of complications.

Single-incision laparoscopy surgery (SILS) is used in veterinary medicine for different surgical procedures [17,31,32,33]. Analyzing the data reported in the literature, the time consumed during laparoscopic surgery is evident, especially due to the loss of triangulation and instrument collisions [34]. The technique does not need organ retraction and allows for an excellent visualization of the adrenal gland. In human medicine, it is routinely used for the removal of adrenal masses [35,36].

In veterinary medicine, only one study has been published, and it was limited to researching and evaluating the feasibility of the technique [12,37]. Some authors have advocated the feasibility of this procedure in veterinary medicine; however, currently, no clinical studies have been published [3].

The study had some limitations. The group of dogs involved in the research was heterogeneous and consisted of animals with various histologic types of adrenal gland tumors and with different clinical presentations. Analyzing subgroups of dogs with homogenous characteristics might have revealed more precise information regarding complication rates and the outcomes of specific tumor types. The small number of dogs involved in the study is another limitation, and additional research involving multiple specialist veterinary centers could improve the case study by enabling the comparison of different techniques to determine which is more advantageous for the patient and the surgeon.

Another main limitation was the inclusion of only four right-sided lesions compared with the twelve left-side lesions. Greater sample homogeneity or more comparisons between two similar groups would be beneficial in clarifying and highlighting the differences in the technique regarding the two glands.

## 5. Conclusions

Based on this primary experience, a two-port laparoscopic adrenalectomy is a safe and feasible procedure for selected patients with adrenal tumors when performed by a surgeon experienced in laparoscopic and adrenal surgery. However, more surgical data regarding this technique are required to confirm the preliminary study impressions.

## Figures and Tables

**Table 1:** The signalment, clinical and laboratory findings, ultrasound size of the left side (LS) and right-side adrenal tumors (AT), and other ultrasound abnormalities of the dogs included in the study and LDDS test results presented in the table.

Patient	Signalment	Clinical Signs	Laboratory Findings	Ultrasound Findings	LDDS Test Results
1	11y, NF, 37 Kg, German shepherd	PU/PD, alopecia, polyphagia	-	30 × 40 mm LS, mild hepatomegaly	T0: 12.4 mg/dL T4: 16.3 mg/dL T8: 11.5 mg/dL
2	8y, NM, 40 Kg, German shepherd	Cutaneous calcinosis, alopecia	ALP 4512 U/L	34 × 40 mm RS, mild hepatomegaly	T0: 3.2 mg/dL T4: 3.1 mg/dL T8: 4.5 mg/dL
3	9y, NF, 38 Kg, Golden retriever	PU/PD, polyphagia, alopecia, thin skin	WBC 22 × 10^10^/L ALP 328 U/L TCHOL 3g/L	25 × 40 mm LS	T0: 4.6 mg/dL T4: 5.7 mg/dL T8: 5.4 mg/dL
4	8y, NM, 37 Kg, Golden retriever	Incidental finding	-	33 × 33 mm LS, prostatic cysts, mild hepatomegaly	T0: 2.7 mg/dL T4: <1.0 mg/dL T8: <1.0 mg/dL
5	11y, NM, 27 Kg, Mixed breed	PU/PD; hematuria, stranguria, astenia	WBC 18 × 10^10^/L ALP 2907 U/L	25 × 35 mm RS	T0: 3.4 mg/dL T4: 3.4 mg/dL T8: 3.8 mg/dL
6	9y, NF, 14 Kg, Mixed breed	PU/PD	-	34 × 40 mm LS	T0: 5.5 mg/dL T4: 5.4 mg/dL T8: 6.5 mg/dL
7	12y, N, 9 Kg, Mixed breed	Incidental finding	ALP 4712 U/L	35 × 40 mm LS, hepatomegaly, reduction cortical medullary ratio	T0: 8.9 mg/dL T4: 7.4 mg/dL T8: 6.4 mg/dL
8	9y, M, 10 Kg, Yorkshire terrier	PU/PD	-	24 × 30 RS	T0: 9.3 mg/dL T4: 9.0 mg/dL T8: 8.7 mg/dL
9	9y, NF, 13 Kg, Yorkshire terrier	Incidental finding	-	24 × 40 mm LS, mild hepatomegaly	T0: 4.3 mg/dL T4: 6.5 mg/dL T8: 6.2 mg/dL
10	12y, NF, 9 Kg, Poodle	PU/PD, polyphagia, alopecia	ALP 3223 U/L ALT 340 U/L	23 × 33 mm RS, mild hepatomegaly	T0: 4.8 mg/dL T4: 3.6 mg/dL T8: 4.2 mg/dL
11	10y, M, 10 Kg, Cocker spaniel	PU/PD, calcinosis cutis, alopecia	WBC 20 × 10^10^/L ALP 2858 U/L ALT 437 U/L	23 × 30 mm LS	T0: 15.5 mg/dL T4: 13.1 mg/dL T8: 10.9 mg/dL
12	9y, NF, 12 Kg, Cocker spaniel	PU/PD, calcinosis cutis, alopecia	ALP 2850 U/L	32 × 35 mm LS	T0: 9.7 mg/dL T4: 8.4 mg/dL T8: 8.9 mg/dL
13	9y, 27 Kg, Epagneul Breton	Incidental finding	-	28 × 38 mm LS	T0: 5.5 mg/dL T4: <1.0 mg/dL T8: <1.0 mg/dL
14	6y, 18 Kg, English bulldog	PU/PD; hematuria, calcinosis cutis	ALP 4340 U/L	36 × 30 mm LS	T0: 7.7 mg/dL T4: 6.6 mg/dL T8: 6.5 mg/dL
15	7y, 33 Kg, Weimaraner	Incidental finding	-	34 × 40 mm LS	T0: 2.6 mg/dL T4: <1.0 mg/dL T8: <1.0 mg/dL
16	12y, NF, 9 Kg, Dachshund	PU/PD	WBC 18 × 10^10^/L ALP 3858 U/L ALT 473 U/L	24 × 40 mm LS	T0: 3.1 mg/dL T4: 2.9 mg/dL T8: 3.7 mg/dL

**Table 2:** In the table are reported data about intraoperative and postoperative complications, surgical time, outcome, and histological diagnosis collected in the study population.

Dog	Breed	Intraoperative Complications	Postoperative Complications	Surgical Time (Min)	Outcome	Histological Diagnosis
1	German shepherd	-	-	100	Alive	Carcinoma
2	German shepherd	-	-	120	Alive	Carcinoma
3	Golden retriever	Capsule rupture	-	85	Lost follow-up	Carcinoma with vascular emboli
4	Golden retriever	-	-	100	Alive	Adenoma
5	Mixed breed	-	-	85	18 months, euthanasia for oral melanoma	Carcinoma
6	Mixed breed	-	-	80	12 months	Carcinoma with vascular emboli
7	Mixed breed	-	Periportal cellulitis	74	Alive	Carcinoma
8	Yorkshire terrier	-	-	40	6 months	Carcinoma with vascular emboli
9	Yorkshire terrier	-	-	50	Lost follow-up	Adenoma
10	Poodle	-	-	74	4 months	Carcinoma with vascular and lymphatic emboli
11	Cocker spaniel	Capsule rupture	Periportal cellulitis	80	8 months	Carcinoma with vascular and lymphatic emboli
12	Cocker spaniel	Capsule rupture	Periportal cellulitis	55	14 months	Carcinoma
13	Epagneul breton	-	-	35	Alive	Carcinoma
14	English bulldog	Capsule rupture	Mild peritonitis, periportal cellulitis	60	3 years, metastasis	Carcinoma
15	Weimaraner	-	-	40	Alive	Adenoma
16	Dachshund	Capsule rupture	-	50	5 years, metastasis	Carcinoma

## Data Availability

The data generated in this study are in the tables of this article. For any further information, the reader can contact the authors.
